# Conditionally immortalized stem cell lines from human spinal cord retain regional identity and generate functional V2a interneurons and motorneurons

**DOI:** 10.1186/scrt220

**Published:** 2013-06-07

**Authors:** Graham Cocks, Nataliya Romanyuk, Takashi Amemori, Pavla Jendelova, Oksana Forostyak, Aaron R Jeffries, Leo Perfect, Sandrine Thuret, Govindan Dayanithi, Eva Sykova, Jack Price

**Affiliations:** 1 The James Black Centre, Department of Neuroscience, King’s College London, 125 Coldharbour Lane, London, UK; 2 Institute of Experimental Medicine, ASCR, Prague, Czech Republic; 3 Department of Neuroscience, 2nd Faculty of Medicine, Charles University, Prague, Czech Republic; 4 Institut National de la Santé et de la Recherche Médicale, Unité de recherche U710, Montpellier and Ecole Pratique des Hautes Etudes, Université Montpellier 2, Paris F-75007, France

**Keywords:** Neural stem cells, Spinal cord, V2a interneurons, Motoneurons, Voltage-operated Ca^2+^ channels, Spontaneous Ca^2+^ oscillations

## Abstract

**Introduction:**

The use of immortalized neural stem cells either as models of neural development *in vitro* or as cellular therapies in central nervous system (CNS) disorders has been controversial. This controversy has centered on the capacity of immortalized cells to retain characteristic features of the progenitor cells resident in the tissue of origin from which they were derived, and the potential for tumorogenicity as a result of immortalization. Here, we report the generation of conditionally immortalized neural stem cell lines from human fetal spinal cord tissue, which addresses these issues.

**Methods:**

Clonal neural stem cell lines were derived from 10-week-old human fetal spinal cord and conditionally immortalized with an inducible form of *cMyc*. The derived lines were karyotyped, transcriptionally profiled by microarray, and assessed against a panel of spinal cord progenitor markers with immunocytochemistry. In addition, the lines were differentiated and assessed for the presence of neuronal fate markers and functional calcium channels. Finally, a clonal line expressing eGFP was grafted into lesioned rat spinal cord and assessed for survival, differentiation characteristics, and tumorogenicity.

**Results:**

We demonstrate that these clonal lines (a) retain a clear transcriptional signature of ventral spinal cord progenitors and a normal karyotype after extensive propagation *in vitro*, (b) differentiate into relevant ventral neuronal subtypes with functional T-, L-, N-, and P/Q-type Ca^2+^ channels and spontaneous calcium oscillations, and (c) stably engraft into lesioned rat spinal cord without tumorogenicity.

**Conclusions:**

We propose that these cells represent a useful tool both for the *in vitro* study of differentiation into ventral spinal cord neuronal subtypes, and for examining the potential of conditionally immortalized neural stem cells to facilitate functional recovery after spinal cord injury or disease.

## Introduction

Stem cells have received substantial interest both for their potential as *in vitro* tools to study development and as potential therapeutic agents in a range of degenerative diseases of the nervous system [1]. One area of particularly strong research activity has been spinal cord injury (SCI), for which treatment options are very limited [2]. Stem cells derived from a range of different tissue sources and developmental stages have been studied for their capacity to elicit functional recovery in animal models of SCI [3,4]. One such approach has been to generate immortalized neural stem cell lines from postmortem human fetal spinal cord tissue for transplantation [5-8]. An important question in the use of tissue-specific immortalized neural stem cell lines as cellular therapies is the extent to which these cells are able to retain the phenotypic characteristics of the tissue of origin after immortalization, prolonged *in vitro* propagation, and engraftment into lesioned tissue.

In the current study, we generated three clonal neural stem cell lines from human fetal spinal cord, designated SPC-01, SPC-04, and SPC-06, conditionally immortalized with 4-hydroxy tamoxifen (4-OHT)-inducible cMyc (cMycER^TAM^) [9]. This technology involves transducing primary dissociated cells with a retrovirus containing the gene *cMyc* fused to a mutated form of the estrogen receptor. This fusion protein is specifically activated by the presence of the synthetic ligand 4-OHT, triggering dimerization and translocation to the nucleus. The nuclear cMycER protein regulates gene expression, and in particular, directly upregulates telomerase [10], thus allowing the cell to proliferate indefinitely without undergoing replicative senescence. Removal of 4-OHT from the media results in inactivation of cMycER and terminal cellular differentiation [11].

To assess whether these conditionally immortalized neural stem cells retain the identity of their tissue of origin after prolonged *in vitro* propagation, we performed a genome-wide transcriptome analysis. This dataset was then analyzed in terms of the expression of homeodomain transcription factors known to play an instructive role in the identity of progenitor subtypes in the developing spinal cord [12], and the findings validated by immunostaining. The ventral spinal cord has four major interneuron progenitor subdomains (p0, p1, p2, and p3), and one motoneuron progenitor subdomain (pMN) specified by the cross-repressive activities of class I and II homeodomain transcription factors [12]. The ventral p2 domain of the spinal cord, comprising Nkx6.1^+^/Irx3^+^ cells, gives rise to two main lineages of interneurons designated V2a and V2b, specified by differential Notch signaling [13,14]. A third lineage designated V2c, derived from the V2b lineage and dependent on Sox1 expression, has also recently been identified [15]. The genome-wide transcriptome analysis of the conditionally immortalized neural stem cell lines reported here, and subsequently confirmed by immunostaining, revealed a homeodomain transcription-factor profile indicative of the ventral spinal cord p2 and pMN domains.

Furthermore, we demonstrated that on removal of growth factors and 4-OHT, these cells differentiate into V2 interneurons and motoneurons, consistent with the expression of p2 and pMN domain markers in the progenitor cells.

To study the functional properties of neurons derived from these conditionally immortalized neural stem cells, we assessed the Ca^2+^ responses induced by high K^+^ and specific Ca^2+^ channel blockers. Intracellular Ca^2+^ changes control many neuronal functions including neurotransmitter release [16], membrane excitability [17], gene transcription [18], and growth [19]. It was shown previously that, during the period of synaptogenesis, acutely dissociated embryonic motoneurons express a great variety of voltage-operated Ca^2+^ channels (VOCCs), able to induce a Ca^2+^-induced Ca^2+^ release (CICR) through a new type of intracellular Ca^2+^ pathway functionally linked to P-type Ca_v_2.1 Ca^2+^ channel subunits [20-22]. We found that neurons derived from the clonal lines described here express functional T-, L-, N-, and P/Q-type Ca^2+^ channels. Furthermore, we demonstrated that a subset of these neurons exhibit spontaneous calcium oscillations typically observed in dissociated embryonic rat motoneurons cultures [23].

Finally, in a series of grafting experiments into lesioned rat spinal cord, we demonstrated that these cells are able to stably engraft, differentiate into choline acetyltransferase positive (ChAT^+^) motoneurons, and show robust survival after 4 months without tumorogenicity.

## Materials and methods

### Generation of clonal lines

Ten-week-old fetal tissue was obtained from Advanced Bioscience Resources (Alameda, CA, USA) after normal terminations and in accordance with nationally (UK and USA) approved ethical and legal guidelines [24,25]. Primary cells were prepared by finely chopping the cervical region of the fetal spinal cord with a scalpel and dissociation at 37°C with 0.25% trypsin (BioWhittaker) in DMEM:F12 (Gibco), followed by 0.25 mg/ml soybean trypsin inhibitor (Gibco). Clonal conditionally immortalized cell lines were generated by using MMLV-type retrovirus encoding the gene cMYC-ER^TAM^, as previously described [9,11]. In brief, primary spinal cord cells transduced with cMYC-ER^TAM^ were plated at clonal density, and individual colonies were passaged by using glass cloning cylinders (Sigma-Aldrich). Spinal cord clones SPC-01, SPC-04, and SPC-06 were initially selected based on uniform growth over 20 population doublings before further characterization. A version of spinal cord clonal line 1 (SPC-01) expressing eGFP was also generated by using a lentiviral vector containing a ubiquitous chromatin opening element to prevent silencing on engraftment, as previously described [26].

### Cell growth and differentiation

Cell lines were routinely cultured, as previously described in Pollock *et al*. [11]. In brief, cells were grown on laminin-coated (Sigma-Aldrich), tissue-culture flasks in DMEM/F12 supplemented with bFGF (10 ng/ml), EGF (20 ng/ml) (PeproTech, UK); human serum albumin (0.03%) (Baxter Healthcare); L-glutamine (2 m*M*) (Gibco); human transferrin (100 μg/ml), putrescine dihydrochloride (16.2 μg/ml), human insulin (5 μg/ml), progesterone (60 ng/ml), sodium selenite (selenium) (40 ng/ml), and 4-OHT (100 n*M*) (Sigma-Aldrich). Cell differentiation was triggered by the removal of growth factors and 4-OHT from the media with or without the addition of *N*-[*N*-(3,5-difluorophenacetyl)-L-alanyl]-*S*-phenylglycine *t*-butyl ester (DAPT) (10 μ*M*) or all-*trans* retinoic acid (ATRA), as indicated in the text. Long-term growth and population doublings were monitored by recording the total number of cells at each passage.

### Karyotyping analyses

Cells at 70% to 80% confluence were treated with 100 ng/ml colcemid (Life Technologies), for 3 hours, and subjected to hypotonic lysis in 0.075 *M* potassium chloride for 10 minutes at 37°C. Samples were then fixed in methanol/glacial acetic acid (ratio 3:1) and stained with Giemsa on glass slides for analysis.

### Immunocytochemistry

Cells were fixed in 4% paraformaldehyde for 15 minutes at room temperature, washed with PBS, and permeabilized with 0.1% Triton-X100/tris-buffered saline (TBS) for 30 minutes. Nonspecific binding was then blocked with 10% normal donkey serum in TBS for 1 hour at room temperature. Cells were then probed with primary antibodies to Nestin (1:500; Chemicon), Sox2, and ChAT (1:1,000; Millipore), βIII tubulin and Irx3 (1:1,000; Sigma-Aldrich), Tau and S100Β (1:2,000; DAKO), Olig2, and MASH1 (1:200; Millipore), Pax6, Nkx6.1, Lhx3, En1, and Isl1 (all Developmental Studies Hybridoma Bank (University of Iowa), 1:200), and Chx10 and GATA3 (1:250; Abcam) at 4°C overnight. Secondary antibodies used were donkey anti-mouse Alexa 488, donkey anti-rabbit Alexa 594, donkey anti-sheep Alexa 488, donkey anti-goat Alexa 488, and donkey anti-rabbit Alexa 680 (all 1:300, Molecular Probes), as appropriate, in 1% NDS/TBS for 1 hour at room temperature. Cells were then washed with TBS and counterstained with 1 μ*M* Hoechst 33342 (Sigma-Aldrich).

### Cell counting

All cell counting was carried out in biologic triplicate, in which the experiment was replicated with cells plated several passages apart. Each biologic replicate consisted of three wells (technical replicates) for each condition. Each technical replicate consisted of three randomly placed nonoverlapping images taken per well with the 40× objective. Images were imported into ImageJ, and nuclei and target-positive cells were counted manually. The three-well images were averaged to generate one technical replicate. The three technical replicates (wells) were averaged to generate one biologic replicate (plate), which was then used for statistical analysis. All cell counting was carried out from images taken from the 1×70 inverted microscope (Olympus) and processed with the Axio Vision Digital Image Processing Software (Carl Zeiss Inc.). Exposure times were kept consistent for each target. Differences in the proportion of marker-positive cells between cell lines were tested for statistical significance by using a one-way ANOVA for each marker (Prism).

### Microarray analysis

RNA was extracted from cultured cells by using Trizol (Life Technologies), and DNase treated with Turbo DNase (Life Technologies). RNA was assessed for quality with an Agilent Bioanalyzer ensuring an RNA integrity number greater than 9. Genome-wide gene-expression profiling was performed with the Illumina HumanWG-6 v3.0 expression beadchip array, at the Welcome Trust Centre for Human Genetics (Oxford, UK). Sample probe profiles were exported from GenomeStudio into lumi (bioconductor). Variance-stabilizing transformation was applied to datasets, quantile normalized, and log_2_ transformed. All microarray data from this study are available through the Gene Expression Omnibus [27], with accession number GSE37282.

### Drugs and solutions

Unless otherwise stated, all standard chemicals were obtained from Sigma-Aldrich (St. Louis, MO, USA). Sandimmune (Novartis Pharma AG, Basel, Switzerland); Immuran (GlaxoSmith-Kline); Solu-Medrol (Pfizer, Puurs, Belgium); Fura-2 AM 1 m*M* solution in anhydrous DMSO and Pluronic F-127 (30% stock in distilled water) (Molecular Probes, USA); ω-Aga Toxin IVA (ω-Aga IVA), and ω-conotoxin GVIA (ω-GVIA) (Alomone Labs Ltd. Jerusalem, Israel). Concentrated stock solutions of nicardipine were prepared in DMSO, whereas the remaining stock solutions of Ca^2+^-channel toxins were dissolved in dH_2_O. All concentrated stock solutions were stored at −20°C. Test solutions were prepared daily by using aliquots from frozen stocks to obtain the working concentrations. All buffers and solutions used for the Ca^2+^-measurement studies were made by using ion-free dH_2_O from Merck-Germany.

### Solutions for [Ca^2+^]_i_ measurements

Normal Locke (NL) buffer was used for [Ca^2+^]_i_ measurements on single cells in culture, containing (m*M*): NaCl, 140; KCl, 5; MgCl_2_, 1.2; CaCl_2_, 2.2; glucose, 10; HEPES-Tris, 10; BSA, 0.02%; pH 7.25. The osmolarity of the solutions used ranged between 298 and 300 mosmol/l^-1^. High-K^+^ buffer contained (m*M*): NaCl, 90; KCl, 50; MgCl_2_, 1.2; CaCl_2_, 2.2; glucose, 10; HEPES, 10; at pH 7.25. For other K^+^ concentrations, KCl was added at the desired concentration and was adjusted with NaCl appropriately to bring the osmolarity to the required range. The Ca^2+^-channel blockers ω-GVIA and ω-Aga IVA were prepared as concentrated stocks in distilled water, stored at −70°C, and diluted to working concentrations before use. The control and test solutions were applied by using a multiple capillary perfusion system (200 μm inner-diameter capillary tubing; flow rate, 250 μl/min), and the cells were subjected to a constant fast-flow control buffer. Each capillary was fed by a reservoir 30 cm above the bath and connected to a temperature-control device (Harvard-France). In this approach, switching the flow from one capillary to the next resulted in complete solution changes within 2 to 5 seconds. After each application of the tested drug, the cells were washed with control buffer. This method allowed fast and reliable exchange of the solution surrounding the cells.

### [Ca^2+^]_i_ measurements on individual SPC-01-derived neurons

Intracellular calcium ([Ca^2+^]_i_) measurements on single cells were performed after 10 days of differentiation by using fast fluorescence spectrofluorimetry. SPC-01 cells differentiated on 22-mm glass-bottom dishes (WillCo Wells BV) were incubated with 2.5 μ*M* Fura-2 AM plus 0.02% Pluronic F-127 at 24°C for 50 minutes. The preparations were then washed with dye-free solution and kept at 37°C until used. Fluorescence measurements of [Ca^2+^]_i_ were performed with the Zeiss Microscope Photometer System (Fast Fluorescence Photometer (FFP), Zeiss, Germany), based on an inverted microscope (Axiovert; Zeiss) equipped for epifluorescence (objective, Plan-Neofluar 100 ×/1.30 oil immersion). The cells were alternately illuminated (200 Hz) at 340 ± 10 and 380 ± 10 nm. To minimize the background noise of the Fura-2 signal, successive values were averaged to a final time resolution of 308 milliseconds. For fast switching between different excitation wavelengths, a rotating filter wheel was mounted in the excitation light path. A measuring amplifier was synchronized to the filter wheel to measure the fluorescence intensities resulting from different wavelengths. The FFP software controlled the acquisition of intensity data and provided functions for adjusting the signal values, the display and storage of the measured data, and calculations of ion concentrations. A CCD camera was used to visualize the cells. With fluorescence values corrected for background and dark current, [Ca^2+^]_i_ calculations were carried out from the ratio between 340- and 380-nm recordings. Fura-2 calibration was performed with the actual instrument by following the same procedure described previously [28], which yielded R_min_ = 0.16; R_max_ = 3.173; β = 2.968; and K_d_ = 224 at 37°C.

### Statistics

Origin 8.5.1 was used for plotting and statistical procedures (OriginLab). The results are expressed as mean ± SEM. The number of the sample size (*n*) given is the number of cells tested according to the same protocol (control, test drug, recovery) for each group. The figures (traces) show on-line single-cell measurements of the [Ca^2+^]_i_ levels before and after the application of test substances, whereas bar diagrams and numeric data are given as mean ± SEM and present the peak amplitude of the [Ca^2+^]_i_ increase as concentration (in n*M*) calculated fluorescence values of 340/380 nm excitation wavelengths. The results were analyzed by using one-way ANOVA. Differences were considered statistically significant if *P* ≤ 0.05.

### Spinal cord compression lesion and cell transplantation

All animal experiments were approved by the Animal Committee of the Czech Republic and the Animal Care and Use of Animals Committee of the Institute of Experimental Medicine AS CR. Adult male Wistar rats weighing 280 to 300 g were anesthetized with isofluorane vapor inhalation (3% to 5%), and a balloon-induced spinal cord compression lesion was performed at the Th8 to Th9 level of the spinal cord, according to protocols previously described [29]. The animals were assisted with manual urination twice a day until the reflex returned, and gentamicin was administered by intramuscular injection twice a day for 3 days. Cell transplantation was performed 7 days after SCI, according to a previously published procedure [30]. For transplantation, SPC-01 cells were detached by TrypZean (Lonza). Harvested cells were grafted by using a stereotaxic injection instrument (Stoelting Co., Wood Dale, IL, USA) and a nanoinjector pump (KD Scientific Inc, Hillstone, MA, USA). In total, of 5 × 10^5^ cells suspended in 5-μl growth media were injected into the epicenter of the lesion, at a depth of 2 mm. All grafted rats (*n* = 22) were immunosuppressed with Sandimmun (Novartis Pharama AG, Basel, Switzerland; 10 mg/kg intraperitoneally), Immuran (GlaxoSmith-Kline, 4 mg/kg intraperitoneally), and Solu-Medrol (Pfizer, Puurs, Belgium; 2 mg/kg intramuscularly) 24 hours before transplantation and then daily until the end of the experiment.

### Histology and immunohistochemistry

The animals were killed and perfused either 8 weeks (*n* = 16) or 4 months (*n* = 6) after cell transplantation for histologic examination. The rats were deeply anesthetized, and 200 ml of PBS was perfused intracardially into the left ventricle, followed by 300 ml of ice-cold 4% (vol/vol) PFA in 0.1 *M* PBS. The spinal cords were dissected, immersed in 4% (vol/vol) PFA at 4°C for 24 hours, and then placed in 30% (wt/vol) sucrose for 3 days. After freezing, spinal cords were cryosectioned longitudinally in 14-mm-thick slices. To identify SPC-01 cells transplanted into the rat spinal cord, antibodies directed against human mitochondria (MTCO2; mouse monoclonal, 1:125, Abcam), human nuclei (HuNu; mouse monoclonal 1:40, Millipore), choline acetyltransferase ChAT (rabbit polyclonal, 1:100, Abcam), nestin (rabbit polyclonal, 1:200 Millipore), Islet2, Nkx 6.1 (both mouse monoclonal, 1:20, Developmental Studies Hybridoma Bank), and GFAP (mouse monoclonal 1:200, Sigma-Aldrich) were used. The Ki67 index and the number of Nkx 6.1-positive cells were calculated as the ratio of Ki67/HuNu-positive cells or Nkx 6.1/HuNu-positive cells to the total number of HuNu-positive cells.

## Results and discussion

### cMycER conditionally immortalized spinal cord neural stem cells retain a normal karyotype and regional identity after prolonged culture

**Figure 1:**
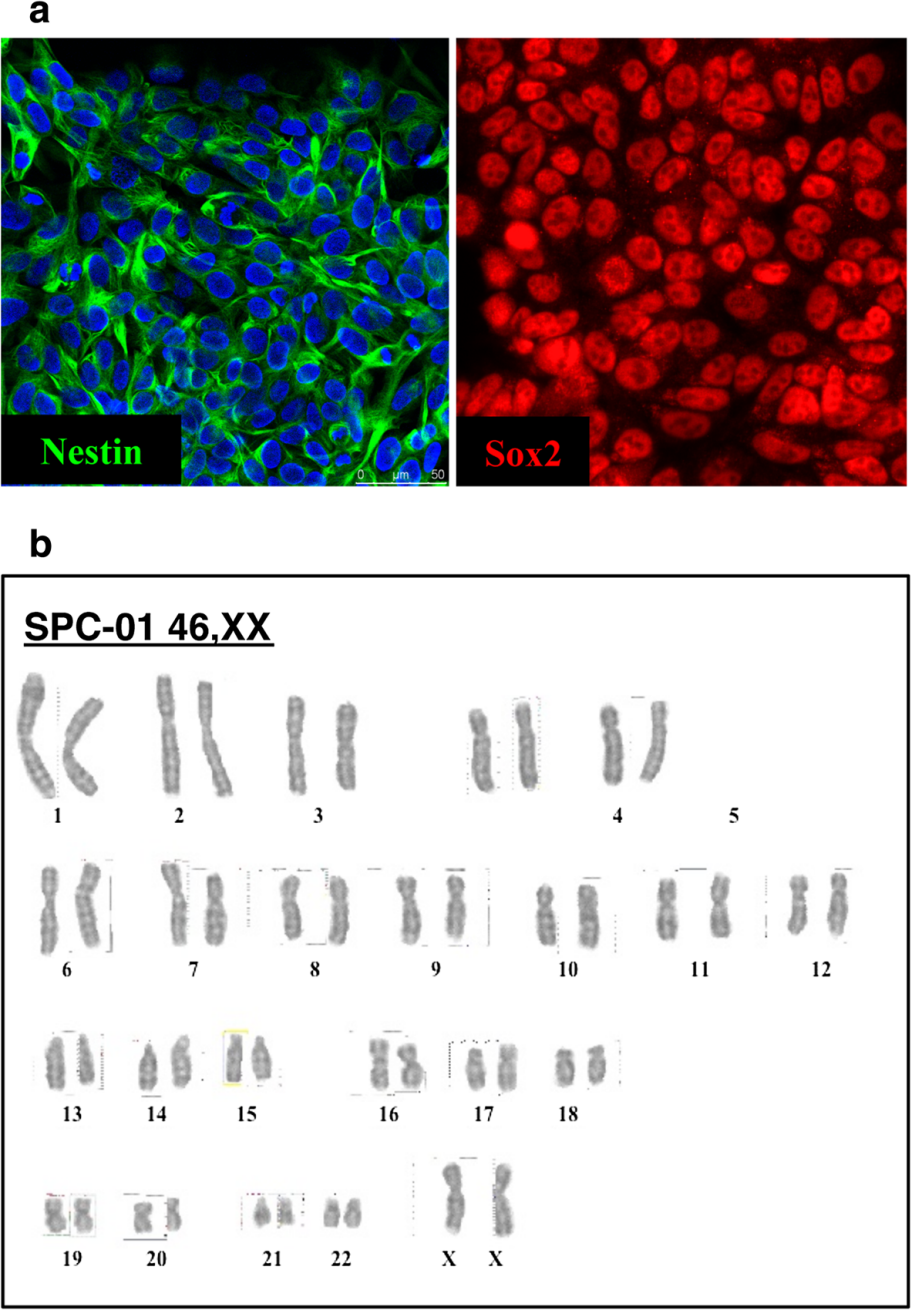
**Characterization of human spinal cord neural stem cells conditionally immortalized with cMycER. (a)** Clonal line SPC-01 expresses the neural stem cell markers Nestin and Sox2. **(b)** Cytogenetic analysis of SPC-01 after 60 population doublings (*n* = 20 metaphase cells), revealed a 46,XX normal female karyotype, demonstrating that conditional immortalization with cMycER and prolonged culture did not affect chromosomal stability in this line.

Three conditionally immortalized clonal lines demonstrating robust growth were characterized and designated spinal cord lines 01, 04, and 06 (SPC-01, SPC-04, and SPC-06). An initial characterization demonstrated that under proliferating conditions, these lines expressed the generic neural stem cell markers NESTIN and SOX2 (Figure 1a and Additional file 1: Figure S1). To examine whether cMyc conditional immortalization and prolonged passage affected genomic integrity, we performed chromosomal analysis on SPC-01 after 60 population doublings. This revealed a 46,XX normal female karyotype in 20 cells analyzed (Figure 1b), demonstrating that these cell lines are karyotypically stable.

**Figure 2:**
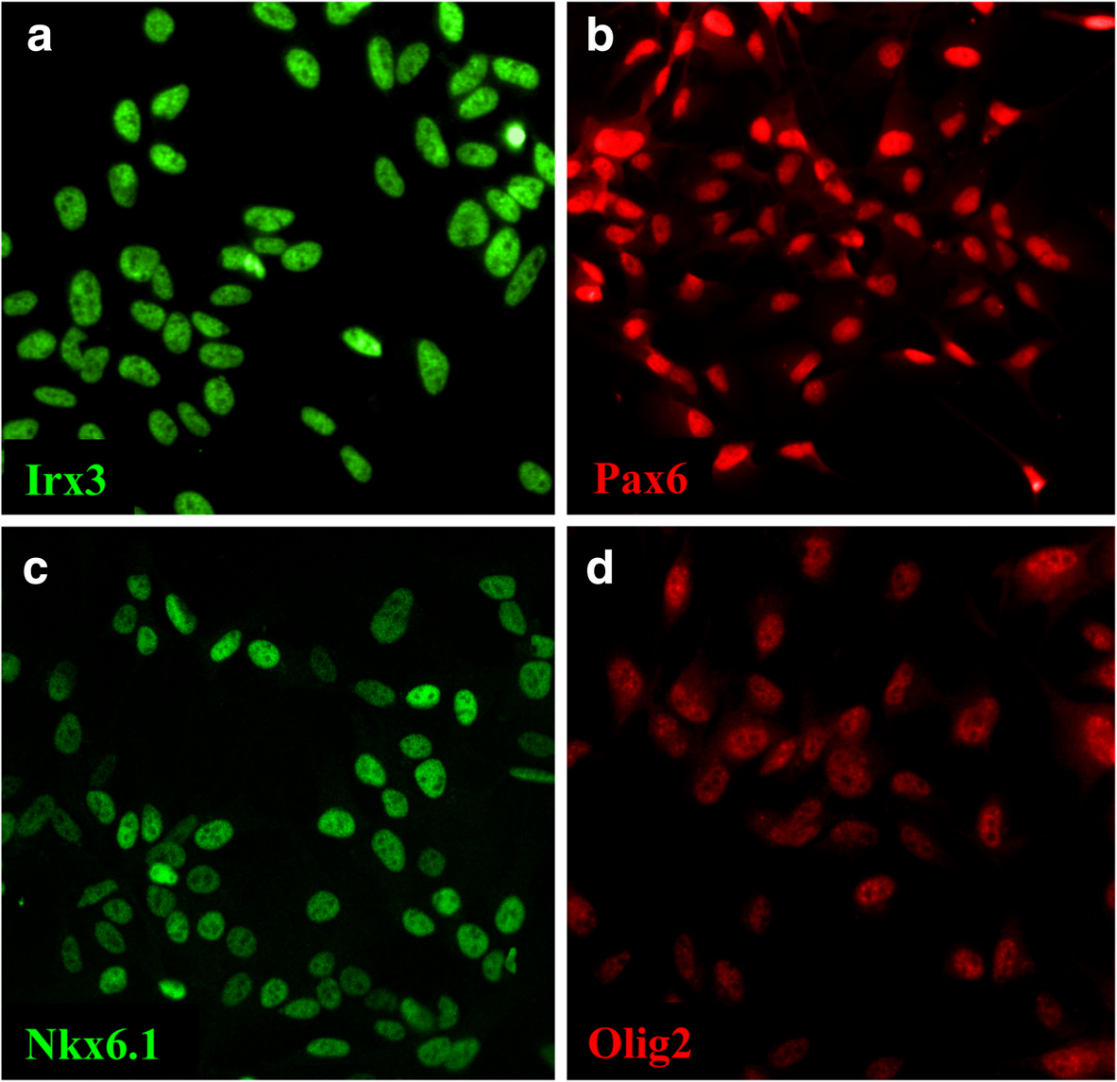
**Immunocytochemistry for ventral spinal cord markers. (a through c)** Undifferentiated SPC-01 cells express the homeodomain transcription factors IRX3, PAX6, and NKX6.1, indicative of the p2 domain of the developing ventral spinal cord. **(d)** A low level of OLIG2 can also be detected in these cells, suggesting a slightly broader developmental potential also encompassing the adjacent pMN domain.

To assess whether the spinal cord lines had retained regional identity, we performed transcriptional profiling by microarray. We identified a subset of homeodomain transcription factors, NKX6.1, NKX6.2, IRX3, and PAX6, to be highly expressed (Table 1), indicative of the developing spinal cord p2 domain [12]. The expression of NKX6.1, IRX3, and PAX6 was confirmed by immunostaining (Figure 2a through c). Expression of the ventral spinal cord transcription factors DBX1, DBX2, NKX2.2, and FOXA2, corresponding to the p0, p1, p3, and floorplate domains, respectively, was below the threshold of detection (Table 1). However, a low level of expression of OLIG2, corresponding to the ventral spinal cord pMN domain, was also observed and confirmed with immunocytochemistry (Figure 2d). The expression of transcription factors corresponding to the roofplate and dorsal spinal cord subdomains LMX1A, GDF7, ATOH1, OLIG3, GSX1, GSX2, and PTF1A were all below the threshold of detection (Table 1). As all three lines had a broadly similar profile of transcription factor expression, we focused all subsequent analysis on SPC-01.

**Table 1:** **Log**_**2**_-**transformed data from Illumina beadchip expression analysis of SPC-01, SPC-04, and SPC-06 cell lines**

		**Log**_**2 **_**expression values (detection P value)**		
**Region**	**Gene ID**	**SPC-01**	**SPC-04**	**SPC-06**
Roof plate	LMX1A	5.55 (0.48)	4.80 (0.30)	4.16 (0.05)
	GDF7	5.94 (0.90)	5.80 (0.89)	5.56 (0.75)
Dorsal spinal cord	ATOH1	5.73 (0.68)	5.85 (0.81)	6.08 (0.91)
	OLIG3	6.12 (0.90)	**6.34 (0.96)**	6.06 (0.91)
	GSX1	5.32 (0.36)	5.09 (0.18)	5.37 (0.73)
	GSX2	4.39 (0.03)	5.18 (0.37)	5.40 (0.46)
	PTF1A	5.08 (0.18)	5.00 (0.15)	5.31 (0.34)
	PAX7	**9.13 (1.00)**	**9.02 (1.00)**	**9.27 (1.00)**
Ventral spinal cord	DBX1	5.04 (0.51)	4.84 (0.37)	6.17 (0.94)
	NKX6.2	**12.08 (1.00)**	**11.50 (1.00)**	**14.12 (1.00)**
	DBX2	5.64 (0.87)	4.86 (0.26)	5.85 (0.85)
	NKX6.1	**6.70 (0.98)**	7.29 (0.99*)	**7.05 (0.99)**
	IRX3	**14.04 (1.00)**	**13.77 (1.00)**	**14.63 (1.00)**
	OLIG2	**6.27 (0.95)**	**8.25 (1.00)**	6.28 (0.93)
	PAX6	**10.07 (1.00)**	**10.11 (1.00)**	**10.33 (1.00)**
	NKX2.2	5.56 (0.51)	5.53 (0.63)	5.20 (0.30)
Floor plate	FOXA2	4.40 (0.09)	4.80 (0.07)	4.37 (0.02)

Expression of a subset of dorsal and ventral spinal cord markers is shown along with detection P values. Detection P values shown in bold represent significant detection above background noise (*P* < 0.05).

### SPC-01 generates V2a interneurons and motoneurons

**Figure 3:**
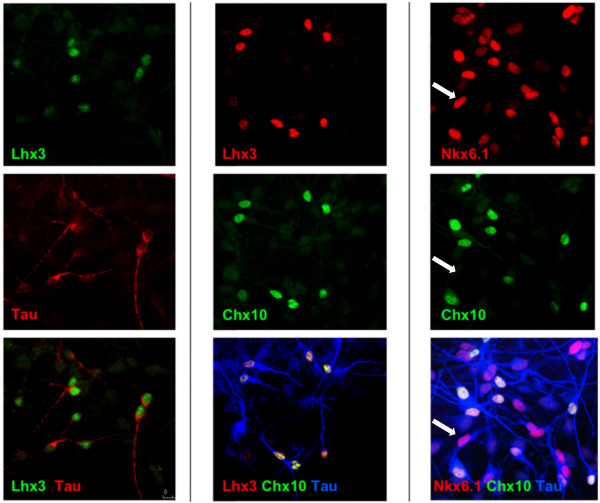
**Characterization of SPC-01 differentiation.** Differentiation of SPC-01 by removal of growth factors and 4-OHT for 7 days gives rise to tau^+^ neurons (left panel). Consistent with the homeodomain transcription-factor profile observed in the undifferentiated cells, a subset of these tau^+^ neurons coexpress LHX3 and CHX10, indicative of V2a interneurons (middle panel). However, a subpopulation of NKX6.1^+^/CHX10^-^ neurons can also be observed (right panel, white arrows).

**Figure 4:**
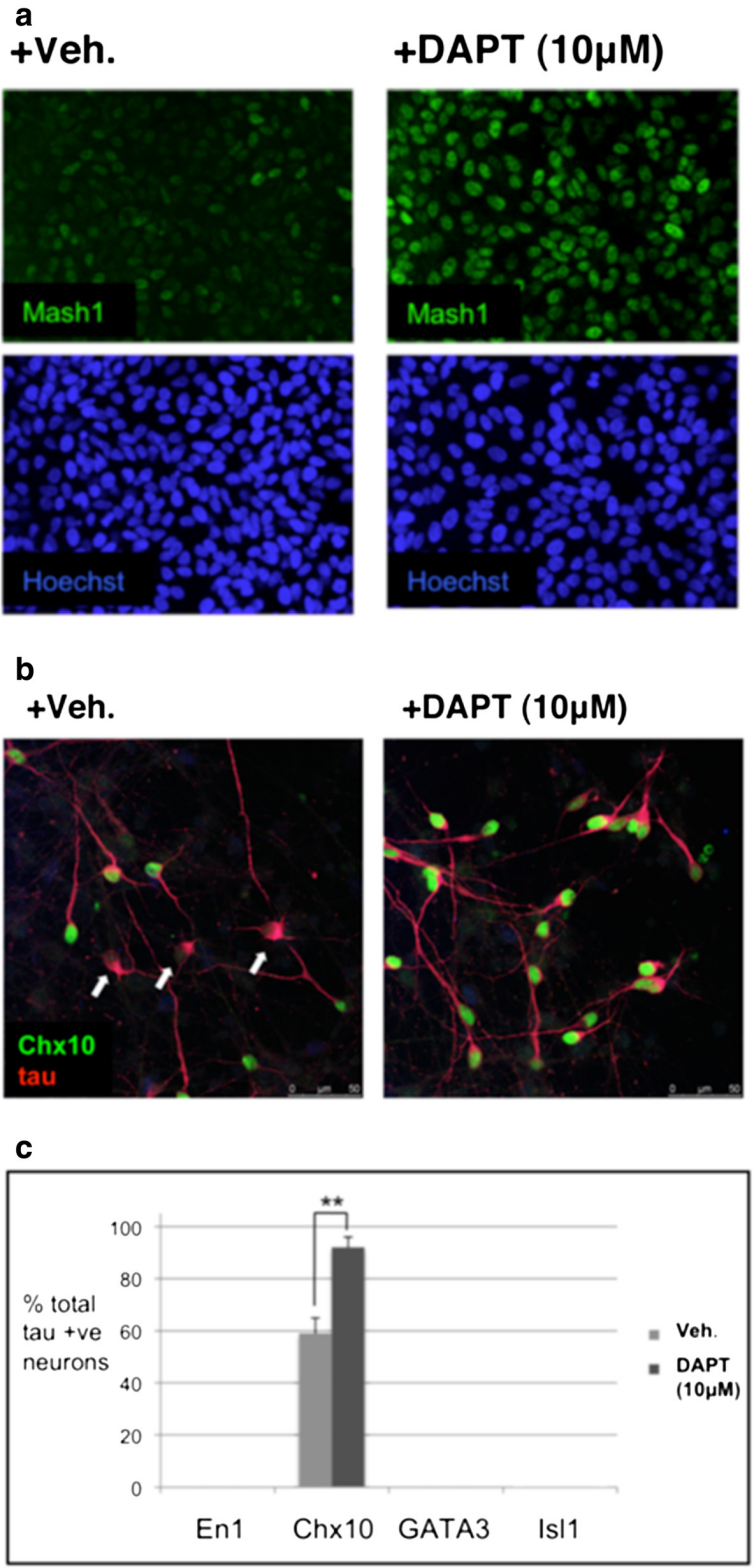
**Notch inhibition regulates the fate of SPC-01. (a)** Treatment of undifferentiated SPC-01 with the γ-secretase inhibitor DAPT (10 μ*M*) for 48 hours upregulates MASH1 expression in SPC-01. **(b, c)** On differentiation, cultures of SPC-01 pretreated with DAPT for 48 hours gave rise to a significantly greater proportion of neurons with a CHX10^+^ V2a interneuronal phenotype (*P* < 0.01, means ± SEM, *n* = 3). This suggests that differential Notch signaling in the undifferentiated SPC-01 cells is giving rise to subpopulations of ventral interneurons, which can be directed into a V2a interneuronal fate by inhibition of Notch.

The expression of ventral spinal cord p2 domain transcription factors in the SPC lines suggests that these lines should give rise to V2 interneurons [31]. After the removal of growth factors and 4-OHT for 7 days, SPC-01 differentiates into a mixed population of mainly neurons and astrocytes (10% and 79%, respectively) with very small numbers of oligodendrocytes (<1%) (Additional file 2: Figure S2). As expected, the majority of neurons generated have an LHX3/CHX10/TAU^+^ fate, indicative of spinal V2a interneurons (Figure 3). Although a majority of neurons generated by SPC-01 after the withdrawal of growth factors and 4-OHT were CHX10^+^, a subset of CHX10-ve/TAU^+^ neurons was found (Figure 3, right panel, and Figure 4b, white arrows). In addition, these CHX10^-^/TAU^+^ neurons were also EN1^-^, GATA3^-^, and ISL1^-^ (Figure 4c), suggesting that they are not V1 or V2b interneurons or motoneurons [31,32]. A recent publication indicated the existence of a third V2 interneuronal subtype termed V2c, generated from V2b Gata3^+^ progenitors [15]. On differentiation, these V2c interneurons upregulate Sox1 while downregulating Gata3. Future work will seek to determine whether these CHX10^-^/Tau^+^ neurons are V2c interneurons.

Differential Notch signaling has been demonstrated to specify the binary-fate decision of p2 progenitors between V2a excitatory and V2b inhibitory interneurons [13,14]. We therefore speculated that inhibition of Notch should drive a V2a excitatory fate in these clonal lines. Inhibition of Notch for 48 hours by the γ-secretase inhibitor DAPT upregulated Mash1 in undifferentiated SPC-01 (Figure 4a) and, on differentiation, resulted in a significant increase in the proportion of Chx10^+^ neurons (Figure 4b and 4c). Notch inhibition therefore drives a V2a interneuronal fate in these cells. Significant progress has been made in identifying the components and stoichiometric interactions of the transcription factor complexes involved in specifying the fate of p2 progenitors into these different interneuron subtypes [33,34]. The ability of SPC-01 to rapidly and reproducibly differentiate into V2a Chx10^+^ interneurons represents a useful tool to study the acquisition of V2 interneuronal fates *in vitro*.

Given that SPC-01 also expresses low levels of OLIG2, a marker of the pMN domain, we asked if these cells were also competent to give rise to motoneurons. It was shown previously that retinoid signaling is required for the specification of motoneuron fate in the ventral spinal cord [35]. We therefore sought to drive the fate of SPC-01 cells along a motoneuron lineage by the addition of 100 n*M* ATRA for the first 2 days of differentiation. We found that SPC-01 did indeed give rise to ISL1^+^ putative motoneurons (Additional file 3: Figure S3a). However, these Isl1^+^ neurons represent only a small subpopulation (<5%) of the total neurons generated (Additional file 3: Figure S3b), suggesting that the default differentiation of these cells is toward a V2 interneuronal fate.

### [Ca^2+^]_i_ responses in SPC-01-derived neurons

**Figure 5:**
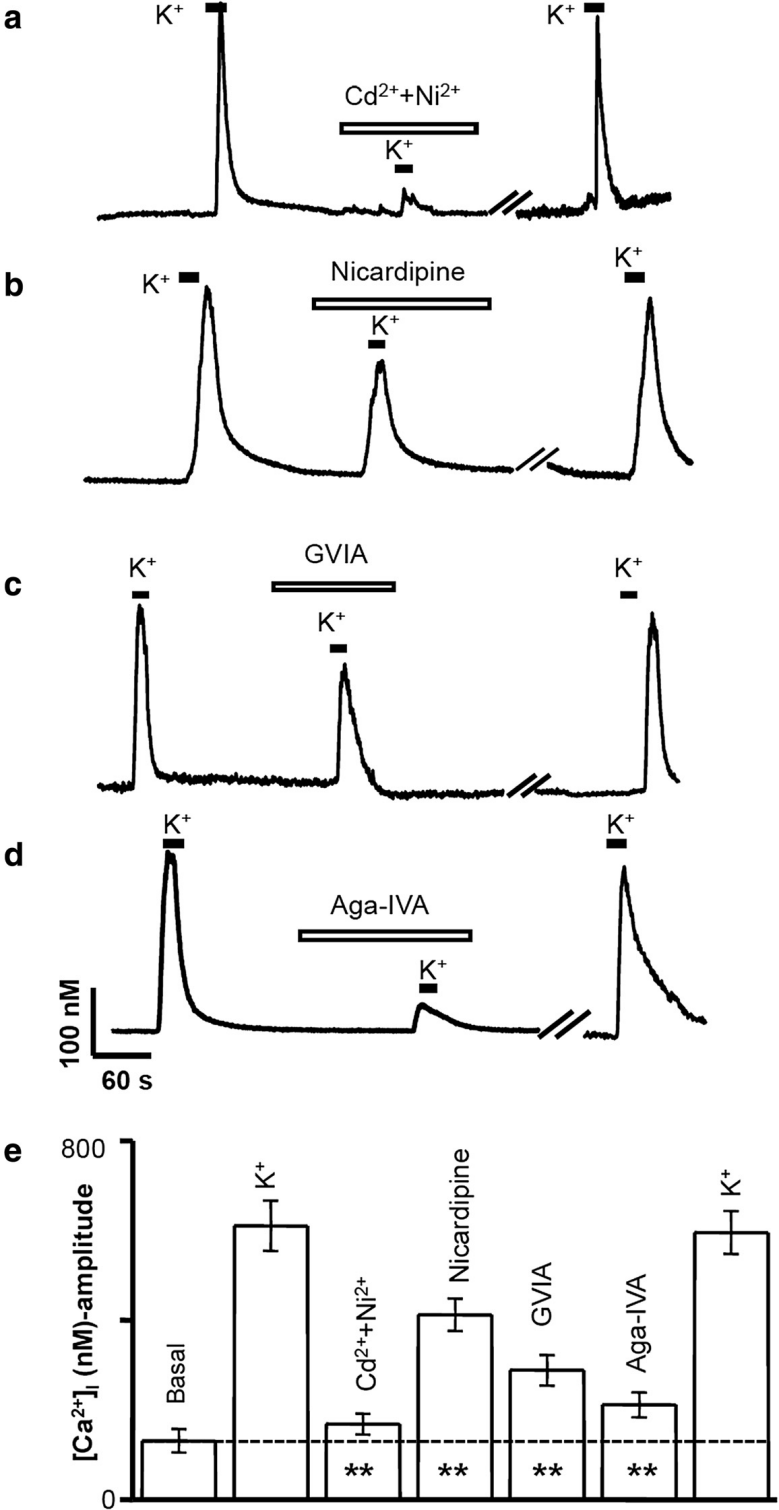
**SPC-01 generates functional neurons. (a)** Preincubation with Cd^2+^ (100 μ*M*) together with Ni^2+^ (50 μ*M*), for 5 minutes significantly reduced the [Ca^2+^]_i_ responses induced by K^+^ in all cells tested by 93% ± 9.2% (*n* = 5), indicating the involvement of voltage-activated Ca^2+^ channels in depolarization-induced Ca^2+^ entry. **(b)** The specific L-type Ca^2+^ channel blocker nicardipine (1 μ*M*) reduced the [Ca^2+^]_i_ responses by 28% ± 7% (*n* = 5; *P* = 0.002). The trace in **(c)** shows the [Ca^2+^]_i_ responses observed in a selected SPC-01-derived neuron induced by 50 m*M* K^+^, preincubated for 2 minutes with Ω -GVIA (800 n*M*) and then stimulated with K^+^. After a washout of 10 minutes, the same cells were subjected to K^+^ to observe recovery. Similarly, the effect of the P/Q-Ca^2+^ channel blocker, Ω -Aga-IVA, 300 n*M* was tested before and during stimulation with high K^+^**(d)**. After washout of the toxin, the [Ca^2+^]_i_ response was shown to be reversible. The histogram **(e)** summarizes the results presented in **(c)** and **(d)**. The resting [Ca^2+^]_i_ level in these cells is indicated as basal. The results are expressed as mean ± SEM; *n* = 4 (Ω-GVIA) and *n* = 5 (Ω-Aga-IVA). Asterisks indicate the statistical significance (*P* > 0.05) versus control K^+^ stimulus.

It was previously shown that Ca^2+^ entering the cytosol via voltage-operated Ca^2+^ channels (VOCCs) regulates many processes in neurons, including the initiation of synaptic transmission [17], gene expression [18], and growth-cone behavior [19]. Although L- and T-type Ca^2+^ currents are found in a wide range of cells, N-, P-, Q-, and R-type Ca^2+^ currents are most prominent in neurons. In purified embryonic rat motoneuron preparations, it has been shown by using the patch-clamp technique that these cells express T-, L-, N-, and P/Q-type Ca^2+^ channels [23]. In the current study, [Ca^2+^]_i_ measurements revealed that SPC-01-derived neurons also express functional T-, L-, N-, and P/Q-type Ca^2+^ channels. Under resting conditions, the basal (resting) [Ca^2+^]_i_ levels in these neurons varied, depending on the day of culture, from 101 ± 12 n*M* at 3 days after plating (*n* = 33) to 113 ± 17 n*M* on the fifth day (*n* = 28). These insignificant variations of the resting [Ca^2+^]_i_ did not appear to have any physiological relevance. Therefore, [Ca^2+^]_i_ measurements were performed on cultures between 3 and 7 days old. After establishing their resting [Ca^2+^]_i_ levels, we determined the functional aspects of SPC-01-derived neurons in culture by evaluating their [Ca^2+^]_i_ responses to high K^+^ (50 m*M* KCl). Only morphologically distinct neuronal-like cells were taken into consideration. We monitored Ca^2+^ entry through VOCCs as [Ca^2+^]_i_ transients evoked by depolarization with 50 m*M* K^+^. The application of high-K^+^ solution evoked a rapid increase in [Ca^2+^]_i_ in 50% (*n* = 56) of the tested cells in three different cultures. Preincubation with Cd^2+^ (100 μ*M*), a nonspecific blocker of high-voltage activated (HVA) Ca^2+^ channels (L-, N-, P-, Q-, and R-types), together with Ni^2+^ (50 μ*M*), a more-specific blocker of low-voltage activated (LVA) Ca^2+^ channels (T-type), for about 5 minutes significantly reduced the [Ca^2+^]_i_ responses induced by K^+^ in all cells tested by 93% ± 9.2% (*n* = 5), indicating the involvement of voltage-activated Ca^2+^ channels in depolarization-induced Ca^2+^ entry (Figure 5a). Further to characterize the involvement of specific subtypes of HVA Ca^2+^ channels, we used specific Ca^2+^ channel blockers, such as nicardipine (for L-type), ω-conotoxin GVIA (for N-type), and ω-Aga toxin IVA (for P/Q- type). The application of 1 μ*M* nicardipine reduced [Ca^2+^]_i_ responses by 28% ± 7% (*n* = 5; *P* = 0.002; Figure 5b), suggesting the contribution of L-type Ca^2+^ channels in SPC-01 neurons. The application of a specific N-type blocker, ω-conotoxin GVIA, at 800 n*M* ω-conotoxin (Figure 5c), reduced the [Ca^2+^]_i_ responses by 42% ± 11% (*P* = 0.001; *n* = 7) in all neurons.

In another set of experiments, we tested the [Ca^2+^]_i_ responses induced by high K^+^ in the presence of the specific P/Q-type Ca^2+^ channel blocker Ω-Aga-IVA. The P/Q-type Ca^2+^ channel is typically expressed in rat embryonic and adult motoneurons [23]. Ω-Aga-IVA (300 n*M*) was found significantly to block the [Ca^2+^]_i_ responses induced by high K^+^ by 76% ± 24% (*n* = 9; P = 0.001; Figure 5d), suggesting the importance of functional P/Q-type Ca^2+^ channels in SPC-01 neurons. These results suggest the expression of T, L-, N- and P/Q-type Ca^2+^ channels in motoneuron-like cells in differentiated SPC-01.

**Figure 6:**
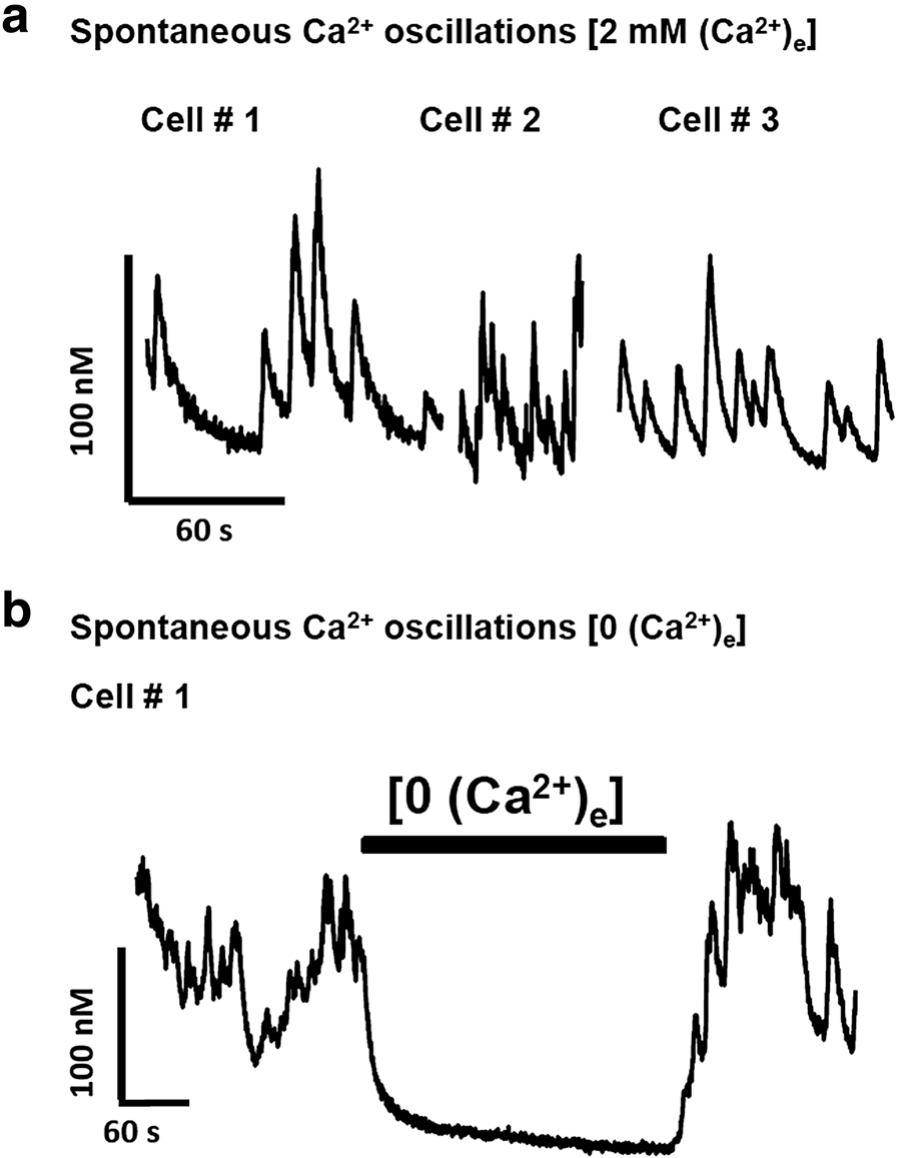
**Differentiated SPC-01 displays spontaneous calcium oscillations.** SPC-01 cells were analyzed in the NL buffer for spontaneous oscillations in [Ca^2+^]_i_ in the presence (Trace A: three examples) or absence of 2 m*M* external Ca^2+^ (Trace B). Note that the Ca^2+^ oscillations were abolished in the absence of external Ca^2+^.

Spontaneous [Ca^2+^]_i_ activity is an essential feature of developing neurons [36]. In the present investigation, we also observed that SPC-01 neurons exhibited spontaneous oscillations in [Ca^2+^]_i_, suggesting the existence of a calcium-induced calcium-release mechanism through the activation of intracellular stores. Twelve of 33 SPC-01 neurons exhibited spontaneous [Ca^2+^]_i_ oscillations under resting conditions (Figure 6a), typically observed in motoneurons from E14 rat cultures [23]. The amplitude of the spontaneous [Ca^2+^]_i_ transients was 296 ± 19, and they appeared with a mean frequency of 1 per 37 ± 6 seconds. These transients were totally abolished by the removal of external Ca^2+^ in all seven tested neurons (Figure 6b). Together, these data provide evidence that SPC-01 generates functional neurons that express multiple Ca^2+^ channels and spontaneous activity characteristic of motoneurons.

### SPC-01 stably engrafts in the lesioned rat spinal cord without tumorogenicity

**Figure 7:**
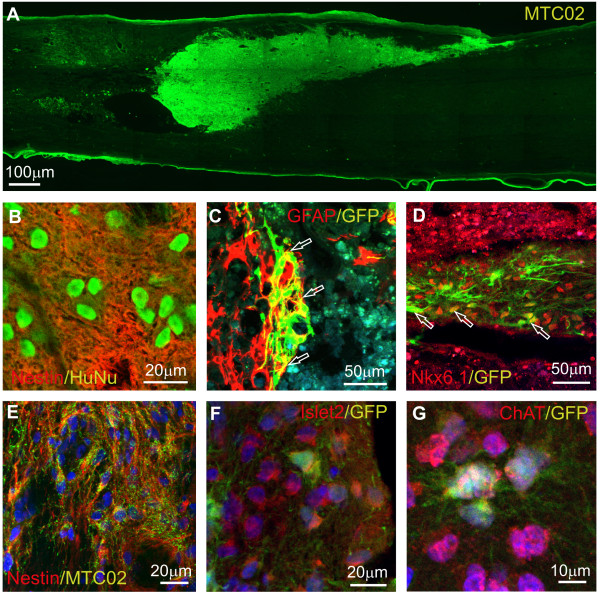
**Engraftment of SPC-01 in lesioned rat spinal cord. (a)** Survival of SPC-01_eGFP was observed 4 months after engraftment into lesioned rat spinal cord. Two months after transplantation, the majority of SPC-01 cells remained nestin-positive **(b)**, and a subpopulation of these also coexpressed GFAP (arrows) **(c)**. Approximately 20% of the grafted cells expressed NKX6.1 (arrows) **(d)**. Four months after transplantation, nestin expression was condensed into long fibers **(e)**, and a subpopulation of cells expressed the motoneuron markers ISL2 **(f)** and ChAT **(g**).

The cMycER^TAM^ conditional immortalization technology has been used to generate neural stem cell lines from different regions of the CNS as potential cellular therapies for conditions including stroke and Parkinson disease [11,37,38]. A cMycER^TAM^ conditionally immortalized human cortical cell line is currently being evaluated in a phase I clinical trial for stroke [39]. We have assessed the capacity of SPC-01 transduced with eGFP to both survive engraftment in the lesioned rat spinal cord and differentiate into appropriate neuronal subtypes without tumorogenicity. Robust engraftment of SPC-01_eGFP was observed 4 months after engraftment (Figure 7a). In all cases, cells filled the lesion cavity and did not migrate far from the lesion. Two months after transplantation, the majority of SPC-01 cells were nestin-positive (Figure 7b), a subpopulation of which also coexpressed GFAP (Figure 7c; see Additional file 4: Figure S4a). Approximately 20% of the grafted cells expressed NKX6.1 (Figure 7d; Additional file 4: Figure S4b). Four months after transplantation, nestin expression was confined to individual fibers (Figure 7e; Additional file 4: Figure S4c), and we observed the expression of the motoneuron markers ISL2 (Figure 7f; Additional file 4: Figure S4d) and ChAT (Figure 7g; Additional file 4: Figure S4e) in a subpopulation of cells. The Ki67 proliferation index of *in vivo* grafted cells ranged from 1.5% to 5.3%, and no tumor formation or hyperproliferative activity was observed during the whole *in vivo* period.

## Conclusions

We generated immortalized neural stem cell lines from human fetal spinal cord; these retain the phenotypic characteristics of the tissue of origin even after prolonged *in vitro* propagation and engraftment into lesioned rodent spinal cord. These cell lines therefore represent a useful tool for studying V2 interneuron differentiation *in vitro* and for further examining the potential of human neural stem cells as cellular therapies for spinal cord injury.

## Abbreviations

4-OHT: 4-hydroxy tamoxifen; ATRA: All-*trans* retinoic acid; ChAT: Choline acetyltransferase; DAPT: *N*-[*N*-(3,5-difluorophenacetyl)-L-alanyl]-*S*-phenylglycine *t*-butyl ester; eGFP: Enhanced green fluorescent protein; NL: Normal Locke buffer; SPC-01: Spinal cord neural stem cell clone 01; VOCCs: Voltage-operated Ca^2+^ channels.

## Competing interests

JP is a consultant to ReNeuron PLC. The other authors declare that they have no competing interests.

## Authors’ contributions

GC generated the cell lines, carried out immunocytochemistry, PCR analysis, karyotyping, and drafting of the manuscript. NR, TA, and PJ carried out grafting studies in rat spinal cord and immunohistochemistry, and assisted with the drafting of the manuscript. OF and GD carried out all calcium recordings and assisted with drafting of the manuscript. AJ assisted with the acquisition and analysis of the microarray data, and LP assisted with cell culture and immunocytochemistry. Both AJ and LP provided a critical appraisal of the manuscript. JP, ES, and ST conceived of the study, participated in its design and coordination, and helped to draft the manuscript. All authors read and approved the final manuscript.

## Supplementary Material

Additional file 1: Figure S1 Clonal lines SPC-04 **(A)** and SPC-06 **(B)** express the neural stem cell markers Nestin and Sox2. Click here for file

Additional file 2: Figure S2 The percentage of tau^+^ neurons, GFAP^+^ astrocytes, and O4^+^ oligodendrocytes 7 days after removal of growth factors and 4-OHT (mean ± SEM, *n* = 3) in clonal line SPC-01. Click here for file

Additional file 3: Figure S3 **(A)** Treatment of SPC-01 with ATRA (100 n*M*) for the first 48 hours of a 14-day differentiation protocol gave rise to small numbers of Isl1^+^ putative motoneurons. **(B)** The percentage of tau^+^ neurons expressing the ventral interneuron fate markers En1, Chx10, GATA3, and the motoneuron marker Isl1 after 48 hours of treatment with ATRA (100 n*M*) and a further 5 days of differentiation without growth factors or 4-OHT (means ± SEM, *n* = 3). Click here for file

Additional file 4: Figure S4 Orthographic projections of engrafted cells where Additional file 4: Figures S4A to S4E correspond to Figure 7C to G, respectively. Click here for file

## References

[B1] Conti L Cattaneo E Neural stem cell systems: physiological players or *in vitro* entities? Nat Rev Neurosci 2010 11 176 187 20107441 10.1038/nrn2761

[B2] Thuret S Moon LD Gage FH Therapeutic interventions after spinal cord injury Nat Rev Neurosci 2006 7 628 643 16858391 10.1038/nrn1955

[B3] Donnelly EM Lamanna JJ Boulis NM Stem cell therapy for the spinal cord Stem Cell Res Ther 2012 3 24 22776143 10.1186/scrt115 PMC3580462

[B4] Willerth SM Neural tissue engineering using embryonic and induced pluripotent stem cells Stem Cell Res Ther 2011 2 17 21539726 10.1186/scrt58 PMC3226288

[B5] Onifer SM Cannon AB Whittemore SR Altered differentiation of CNS neural progenitor cells after transplantation into the injured adult rat spinal cord Cell Transplant 1997 6 327 338 9171165 10.1177/096368979700600315

[B6] Li R Thode S Zhou J Richard N Pardinas J Rao MS Sah DW Motoneuron differentiation of immortalized human spinal cord cell lines J Neurosci Res 2000 59 342 352 10679769 10.1002/(sici)1097-4547(20000201)59:3<342::aid-jnr7>3.0.co;2-z

[B7] Roy NS Nakano T Keyoung HM Windrem M Rashbaum WK Alonso ML Kang J Peng W Carpenter MK Lin J Nedergaard M Goldman SA Telomerase immortalization of neuronally restricted progenitor cells derived from the human fetal spinal cord Nat Biotechnol 2004 22 297 305 14990951 10.1038/nbt944

[B8] Xu G Li X Bai Y Bai J Li L Shen L Improving recovery of spinal cord-injured rats by telomerase-driven human neural progenitor cells Restor Neurol Neurosci 2004 22 469 476 15798365

[B9] Littlewood TD Hancock DC Danielian PS Parker MG Evan GI A modified oestrogen receptor ligand-binding domain as an improved switch for the regulation of heterologous proteins Nucleic Acids Res 1995 23 1686 1690 7784172 10.1093/nar/23.10.1686 PMC306922

[B10] Greenberg RA O’Hagan RC Deng H Xiao Q Hann SR Adams RR Lichtsteiner S Chin L Morin GB DePinho RA Telomerase reverse transcriptase gene is a direct target of c-Myc but is not functionally equivalent in cellular transformation Oncogene 1999 18 1219 1226 10022128 10.1038/sj.onc.1202669

[B11] Pollock K Stroemer P Patel S Stevanato L Hope A Miljan E Dong Z Hodges H Price J Sinden JD A conditionally immortal clonal stem cell line from human cortical neuroepithelium for the treatment of ischemic stroke Exp Neurol 2006 1999 143 155 16464451 10.1016/j.expneurol.2005.12.011

[B12] Briscoe J Pierani A Jessell TM Ericson J A homeodomain protein code specifies progenitor cell identity and neuronal fate in the ventral neural tube Cell 2000 101 435 445 10830170 10.1016/s0092-8674(00)80853-3

[B13] Yang X Tomita T Wines-Samuelson M Beglopoulos V Tansey MG Kopan R Shen J Notch1 signaling influences v2 interneuron and motor neuron development in the spinal cord Dev Neurosci 2006 28 102 117 16508308 10.1159/000090757

[B14] Del Barrio MG Taveira-Marques R Muroyama Y Yuk DI Li S Wines-Samuelson M Shen J Smith HK Xiang M Rowitch D Richardson WD A regulatory network involving Foxn4, Mash1 and delta-like 4/Notch1 generates V2a and V2b spinal interneurons from a common progenitor pool Development 2007 134 3427 3436 17728344 10.1242/dev.005868 PMC6329449

[B15] Panayi H Panayiotou E Orford M Genethliou N Mean R Lapathitis G Li S Xiang M Kessaris N Richardson WD Malas S Sox1 is required for the specification of a novel p2-derived interneuron subtype in the mouse ventral spinal cord J Neurosci 2010 30 12274 12280 20844123 10.1523/JNEUROSCI.2402-10.2010 PMC6633433

[B16] Augustine GJ How does calcium trigger neurotransmitter release? Curr Opin Neurobiol 2001 11 320 326 11399430 10.1016/s0959-4388(00)00214-2

[B17] Katz B Neural transmitter release: from quantal secretion to exocytosis and beyond J Neurocytol 2003 32 437 446 15034246 10.1023/B:NEUR.0000020603.84188.03

[B18] Morgan JI Curran T Stimulus-transcription coupling in the nervous system: involvement of the inducible proto-oncogenes fos and jun Annu Rev Neurosci 1991 14 421 451 1903243 10.1146/annurev.ne.14.030191.002225

[B19] Kater SB Mills LR Regulation of growth cone behavior by calcium J Neurosci 1991 11 891 899 2010811 10.1523/JNEUROSCI.11-04-00891.1991 PMC6575390

[B20] Hivert B Bouhanna S Diochot S Camu W Dayanithi G Henderson CE Valmier J Embryonic rat motoneurons express a functional P-type voltage-dependent calcium channel Int J Dev Neurosci 1995 13 429 436 7484213 10.1016/0736-5748(95)00026-d

[B21] Scamps F Valentin S Dayanithi G Valmier J Calcium channel subtypes responsible for voltage-gated intracellular calcium elevations in embryonic rat motoneurons Neuroscience 1998 87 719 730 9758236 10.1016/s0306-4522(98)00165-1

[B22] Scamps F Roig A Boukhaddaoui H Andre S Puech S Valmier J Activation of P-type calcium channel regulates a unique thapsigargin-sensitive calcium pool in embryonic motoneurons Eur J Neurosci 2004 19 977 982 15009145 10.1111/j.0953-816x.2004.03196.x

[B23] Dayanithi G Mechaly I Viero C Aptel H Alphandery S Puech S Bancel F Valmier J Intracellular Ca2+ regulation in rat motoneurons during development Cell Calcium 2006 39 237 246 16324742 10.1016/j.ceca.2005.10.011

[B24] Britain G Human Tissue Act 2004 London: HMSO

[B25] Boer GJ Ethical guidelines for the use of human embryonic or fetal tissue for experimental and clinical neurotransplantation and research: Network of European CNS Transplantation and Restoration (NECTAR) J Neurol 1994 242 1 13 7897446 10.1007/BF00920568

[B26] Zhang F Frost AR Blundell MP Bales O Antoniou MN Thrasher AJ A ubiquitous chromatin opening element (UCOE) confers resistance to DNA methylation-mediated silencing of lentiviral vectors Mol Ther 2010 18 1640 1649 20588258 10.1038/mt.2010.132 PMC2956914

[B27] Genome-wide expression analysis of spinal cord neural stem cell clones. Gene Expression Omnibus accession number GSE37282 http://www.ncbi.nlm.nih.gov/geo/

[B28] Lambert RC Dayanithi G Moos FC Richard P A rise in the intracellular Ca^2+^ concentration of isolated rat supraoptic cells in response to oxytocin J Physiol 1994 478 275 287 7525943 10.1113/jphysiol.1994.sp020249 PMC1155685

[B29] Urdzíková L Jendelová P Glogarová K Burian M Hájek M Syková E Transplantation of bone marrow stem cells as well as mobilization by granulocyte-colony stimulating factor promotes recovery after spinal cord injury in rats J Neurotrauma 2006 23 1379 1391 16958589 10.1089/neu.2006.23.1379

[B30] Amemori T Jendelová P Růzicková K Arboleda D Syková E Co-transplantation of olfactory ensheathing glia and mesenchymal stromal cells does not have synergistic effects after spinal cord injury in the rat Cytotherapy 2010 12 212 225 20196694 10.3109/14653240903440103

[B31] Ericson J Rashbass P Schedl A Brenner-Morton S Kawakami A van Heyningen V Jessell TM Briscoe J Pax6 controls progenitor cell identity and neuronal fate in response to graded Shh signaling Cell 1997 90 169 180 9230312 10.1016/s0092-8674(00)80323-2

[B32] Karunaratne A Hargrave M Poh A Yamada T GATA proteins identify a novel ventral interneuron subclass in the developing chick spinal cord Dev Biol 2002 249 30 43 12217316 10.1006/dbio.2002.0754

[B33] Joshi K Lee S Lee B Lee JW Lee SK LMO4 controls the balance between excitatory and inhibitory spinal V2 interneurons Neuron 2009 61 839 851 19323994 10.1016/j.neuron.2009.02.011 PMC2848494

[B34] Song MR Sun Y Bryson A Gill GN Evans SM Pfaff SL Islet-to-LMO stoichiometries control the function of transcription complexes that specify motor neuron and V2a interneuron identity Development 2009 136 2923 2932 19666821 10.1242/dev.037986 PMC2723064

[B35] Novitch BG Wichterle H Jessell TM Sockanathan S A requirement for retinoic acid-mediated transcriptional activation in ventral neural patterning and motor neuron specification Neuron 2003 40 81 95 14527435 10.1016/j.neuron.2003.08.006

[B36] Spitzer NC Lautermilch NJ Smith RD Gomez TM Coding of neuronal differentiation by calcium transients Bioessays 2000 22 811 817 10944583 10.1002/1521-1878(200009)22:9<811::AID-BIES6>3.0.CO;2-G

[B37] Stroemer P Patel S Hope A Oliveira C Pollock K Sinden J The neural stem cell line CTX0E03 promotes behavioral recovery and endogenous neurogenesis after experimental stroke in a dose-dependent fashion Neurorehabil Neural Repair 2009 23 895 909 19633272 10.1177/1545968309335978

[B38] Miljan EA Hines SJ Pande P Corteling RL Hicks C Zbarsky V Umachandran M Sowinski P Richardson S Tang E Wieruszew M Patel S Stroemer P Sinden JD Implantation of c-mycER TAM immortalized human mesencephalic-derived clonal cell lines ameliorates behavior dysfunction in a rat model of Parkinson’s disease Stem Cells Dev 2009 18 307 319 18554088 10.1089/scd.2008.0078

[B39] U.S. National Institutes of Health A Phase I Safety Trial of CTX0E03 Drug Product Delivered Intracranially in the Treatment of Patients With Stable Ischemic Stroke http://clinicaltrials.gov/ct2/show/NCT01151124

