# Supplementary figures and images for: Conditionally immortalized stem cell lines from human spinal cord retain regional identity and generate functional V2a interneurons and motorneurons
Source: Stem Cell Res Ther. 2013 Jun 7;4(3):69. doi: 10.1186/scrt220 (PMC3706922; doi:10.1186/scrt220)

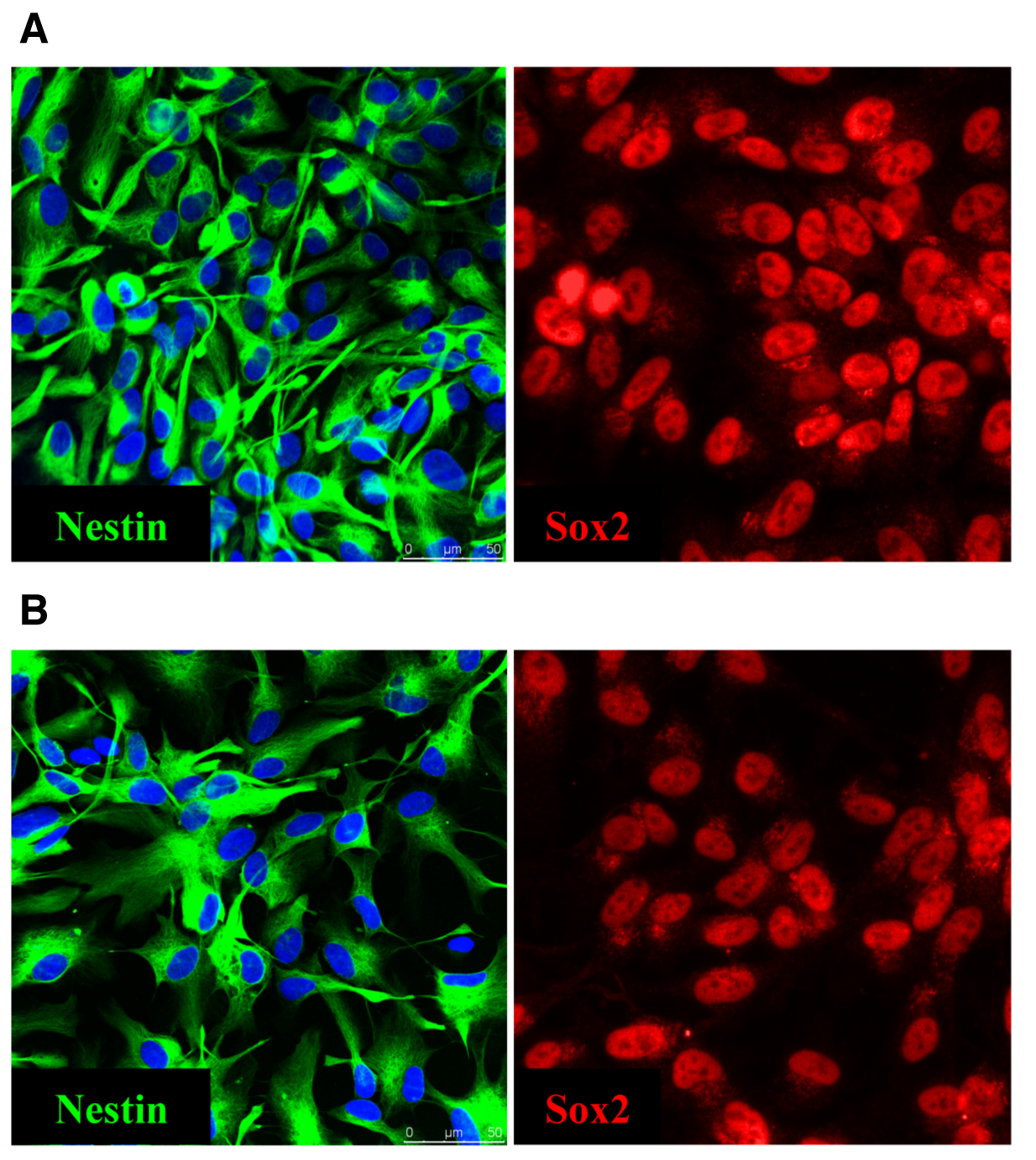

Supplement: Additional file 1: Figure S1 — Clonal lines SPC-04 (A) and SPC-06 (B) express the neural stem cell markers Nestin and Sox2. [file scrt220-S1.tiff]

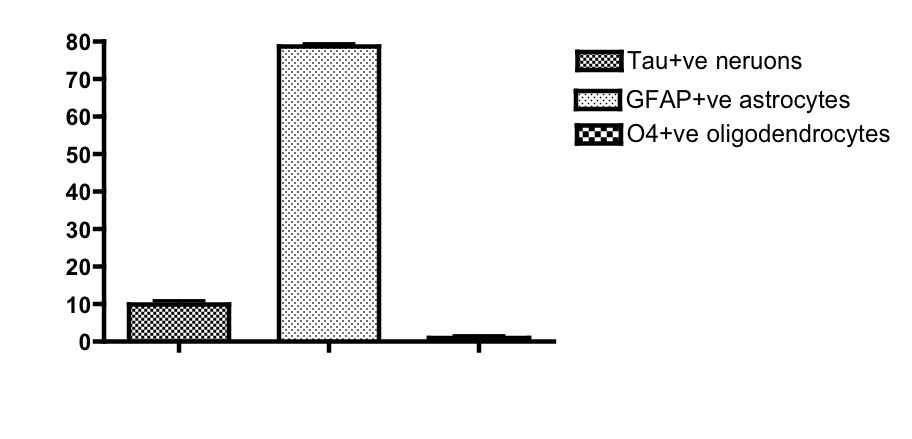

Supplement: Additional file 2: Figure S2 — The percentage of tau+ neurons, GFAP+ astrocytes, and O4+ oligodendrocytes 7 days after removal of growth factors and 4-OHT (mean ± SEM, n = 3) in clonal line SPC-01. [file scrt220-S2.tiff]

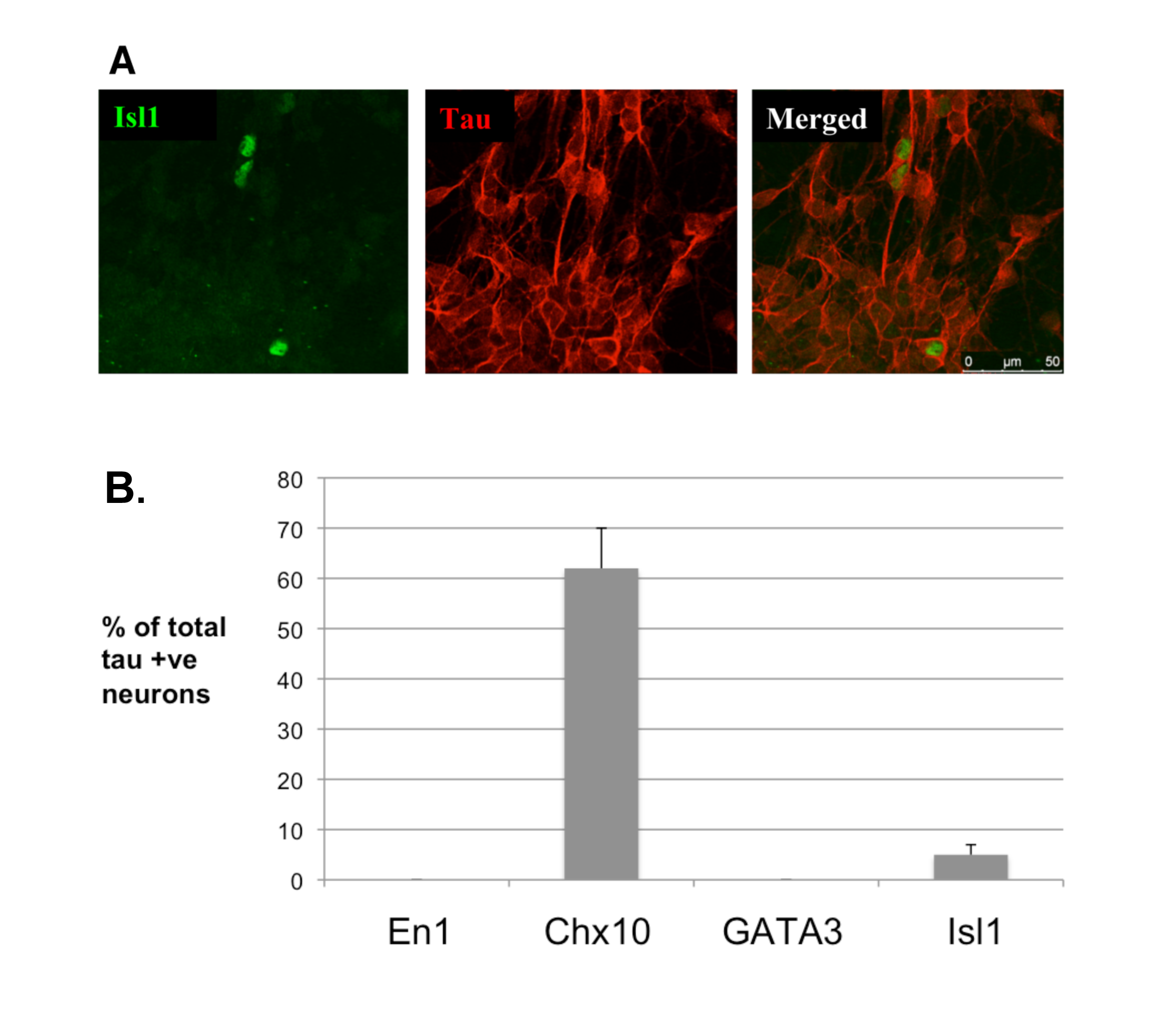

Supplement: Additional file 3: Figure S3 — (A) Treatment of SPC-01 with ATRA (100 nM) for the first 48 hours of a 14-day differentiation protocol gave rise to small numbers of Isl1+ putative motoneurons. (B) The percentage of tau+ neurons expressing the ventral interneuron fate markers En1, Chx10, GATA3, and the motoneuron marker Isl1 after 48 hours of treatment with ATRA (100 nM) and a further 5 days of differentiation without growth factors or 4-OHT (means ± SEM, n = 3). [file scrt220-S3.tiff]

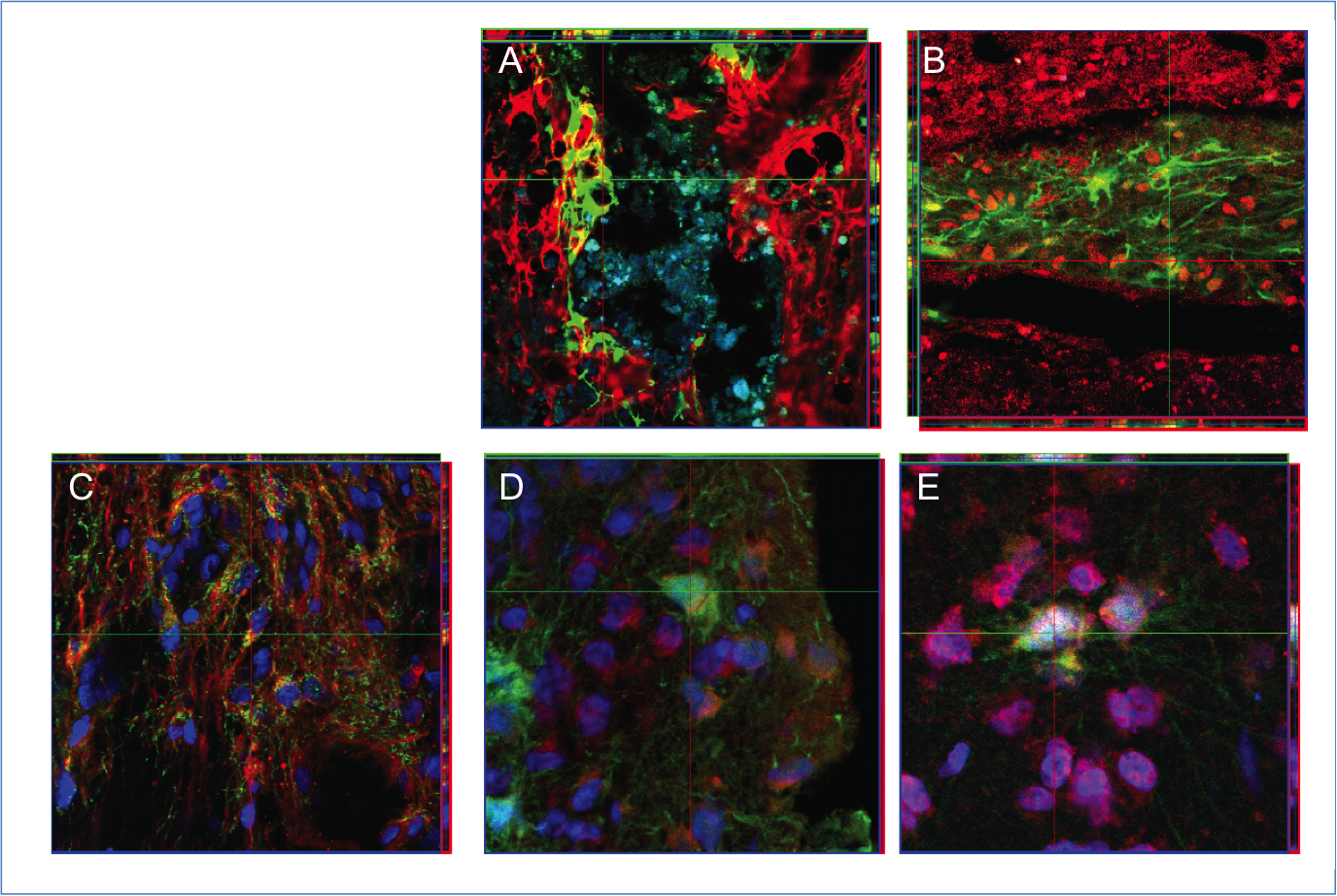

Supplement: Additional file 4: Figure S4 — Orthographic projections of engrafted cells where Additional file 4: Figures S4A to S4E correspond to Figure 7C to G, respectively. [file scrt220-S4.png]
